# The Need for a Preparedness Training Model on Disaster Risk Reduction Based on Culturally Sensitive Public Health Nursing (PHN)

**DOI:** 10.3390/ijerph192416467

**Published:** 2022-12-08

**Authors:** Haris Sofyana, Kusman Ibrahim, Irvan Afriandi, Erna Herawati, Heru Santoso Wahito Nugroho

**Affiliations:** 1 Doctoral Program, Faculty of Medicine, Padjadjaran University, Bandung 45363, Indonesia; 2 Department of Medical and Surgical Nursing, Faculty of Nursing, Padjadjaran University, Bandung 45363, Indonesia; 3 Department of Anthropology, Faculty of Social and Political Sciences, Padjadjaran University, Bandung 45363, Indonesia; 4 Department of Health, Poltekkes Kemenkes Surabaya, Surabaya 60282, Indonesia

**Keywords:** preparedness training, disaster risk reduction, public health nursing, culturally sensitive

## Abstract

The Indonesian Disaster Risk Index (IRBI) in 2018 found that 52.33% of districts or cities in Indonesia were at high risk of natural disasters and the others were at moderate risk. The World Risk Index places Indonesia at number 33 in the very high-risk category. The policy direction of the Implementation of Disaster Management in Indonesia in 2020–2024 is to increase disaster resilience toward sustainable prosperity for sustainable development. Purpose: This study aims to identify the various needs for a culturally sensitive PHN-based disaster risk-reduction preparedness training model. Methods: This study used a descriptive qualitative research design. Data collection was done through in-depth interviews, Focus Group Discussions (FGDs), and expert panel stages in the Indonesian language. Samples involved in the research included 4 experts and 11 informants. Results: There were 10 themes generated from the results. The analysis results revealed that the level of knowledge, attitudes, and skills of the community is still low. Almost all of the people of Mekar Mukti Village stated that they had never received community-based disaster management training. Conclusions: The study findings highlighted the importance of the Disaster Risk-Reduction Preparedness Model Based on Culturally Sensitive Public Health Nursing for the community.

## 1. Introduction

Indonesia is an archipelago prone to disasters. It is located in the path of major earthquake sources such as the megathrust–plate subduction zone and active faults on the mainland. Based on the BNBP report, 295 faults have been identified to be active fault segments that have the potential to produce earthquakes above 6.5 magnitude [1]. Thus, Indonesia’s natural disaster risk is high. The Indonesian Disaster Risk Index (IRBI) in 2018 found that 52.33% of districts or cities in Indonesia were at high risk of natural disasters while the others were at moderate risk. These conditions place Indonesia as one of the countries with the highest rates of natural disasters in the world. Indonesia’s Natural Disaster Risk Index (IRB) statistical data in 2021 for tsunamis, floods, landslides, droughts, and forest fires are also relatively high compared to other countries [2]. 

In 2021, there were 5400 natural disasters in Indonesia, which was an increase from 2500 natural disasters in 2018. This illustrates the very high rate of natural disasters in Indonesia. Floods (1310 incidents), tornadoes (814 incidents), and landslides (633 incidents) are the dominating natural disasters. Natural disasters in 2021 caused more than 8.6 million people to suffer and be displaced, along with the deaths of 676 people. Furthermore, more than 142,000 houses and 3700 education, health, office, road, and bridge facilities were impacted by the natural disasters [3]. 

In addition to natural disasters, Indonesia is also still trying to control the spread of COVID-19 that caused the loss of 100,000 people and has been labeled as a National Non-Natural Disaster [4]. National data on COVID-19 cases, as of 17 March 2022, recorded 153,411 deaths with new confirmed cases of 9528 people, increasing to 5,958,610 people. The COVID-19 pandemic in Indonesia has had an impact on almost all development sectors. One of these development sectors is the Reform (Strengthening) of the Disaster Resilience System, developed to be able to overcome non-natural disasters on a national scale without reducing resilience to handle natural disasters occurring at the same time as non-natural disasters [3]. Data from the Centre For Research On The Epidemiology Of Disasters (CRED) for 2008–2018, revealed that every year Indonesia occupies the top 10 in the world as the country most frequently affected by natural disasters and the country with the highest number of deaths from natural disasters [5]

West Java is one of the areas in Indonesia with a high frequency of natural disasters. 4465 of 5957 villages were included in the category of high-level disaster-prone villages [6]. Additionally, there are numerous active volcanoes in West Java, including Mount Salak, Mount Gede, Mount Tangkuban Parahu, Mount Papandayan, Mount Guntur, Mount Galunggung, and Mount Ciremai. The complex geographical conditions of the West Java region paired with the largest population in Indonesia make this province’s natural disaster risk high. Based on the 2020 Indonesian Disaster Risk Index (IRBI), West Java Province has a risk index of 145.81 (high) [4]. 

This further pushes the direction of the policy for the Implementation of Disaster Management in Indonesia for 2020–2024, which aims to improve disaster resilience toward sustainable prosperity for sustainable development [7]. 

Strengthening the paradigm shift in disaster management from conventional emergency response to disaster risk reduction is one strategy to accomplish this. According to the Disaster Risk Reduction Paradigm (DRR), the community is an active participant in disaster management, thus it must have the necessary knowledge, attitudes, and abilities [8]. This means that in order to effectively manage disasters, the paradigm of disaster risk reduction also calls for community empowerment, which may be accomplished through a variety of community socialization, education, and training programs. 

The study by Setiawan et al. (2017) recommend the importance of training to empower rural communities living in disaster-prone areas to normalize the physical and psychological problems of natural disaster victims [9]. This is in line with the study conducted by Salasa et al. (2017) that identified community preparedness as an important key to minimizing health problems resulting from natural disasters. These results also show that the empowerment process through a contingency planning approach is able to increase youth preparedness against life threatening situations due to disasters. It is advised for all disaster activists to empower youth with contingency planning to increase their readiness to face life threatening situations [10]. In addition, it is necessary to adjust communication strategies to the sociocultural aspects of the local community to effectively emphasize the importance of community empowerment as it would be more easily accepted and relevant to the needs of the people. Culture has become one of the factors for a community’s ability to survive during disasters [11]. 

Other studies have linked this cultural aspect approach with approaches to nursing care for communities affected by disasters. The concept of nursing actions based on cultural considerations provides strong evidence that in a disaster, community nurses must take advantage of cultural forms such as bonds and relationships by providing information and supplements, respecting culture such as local rules and characters as well as healing and comforting the affected residents [12]. In line with this, nurses need to consider the socio-cultural scope of the community and long-term care in order to respond effectively to disasters. This can start from planning, noting positive and negative consequences of assistance, and individual planning in the community [13]. This is in accordance with Senday’s disaster risk reduction framework, which is establishing a national platform for disaster risk reduction. This becomes a general term used as a national policy for coordination and as a policy guide on multisectoral and interdisciplinary disaster risk reduction that involves all relevant entities in a country, whether public or private. Disaster risk reduction requires knowledge, capacity and input from various sectors and organizations, including UN agencies present at the national level. National platforms provide the means to enhance national action to reduce disaster risk, and they represent national mechanisms for the International Disaster Reduction Strategy [14]. Facilitating the community’s ability to anticipate, prepare for and recover from disasters is an important component of the UNISDR strategy for disaster risk reduction [15]. 

One of the theories in nursing that is closely related to community empowerment and cultural care is the theory of the Transcultural Nursing model, published by Madeleine Leininger in 1978. The theory requires an awareness and appreciation of cultural differences that influence providing nursing care with approaches to respecting individual cultural values [16]. Therefore, nurses are required to have knowledge of and engage in a practice that is based conceptually on culture [17]. Transcultural nursing is an area of community and cultural science in the field of nursing that focuses on the differences and similarities between cultures by respecting care, health and illness based on cultural values, beliefs and actions. Thus, several aspects of culture can be studied in an effort to provide nursing care [18]. Based on the background, the research problem is how to create a culturally sensitive disaster risk-reduction model in disaster-prone areas. Therefore, this study aimed to identify the various needs for a culturally sensitive Public Health Nursing (PHN)-based disaster risk-reduction preparedness training model. This study is imperative to explore the need for community preparedness in disaster management based on local culture. 

## 2. Materials and Methods

### 2.1. Study Design

This study used descriptive qualitative research design to identify the various needs for a culturally sensitive Public Health Nursing (PHN)-based disaster risk-reduction preparedness training model. 

The initial quantitative survey had already been conducted in order to identify the model’s demands for training and testing, and to evaluate the model’s effectiveness using a small sample, track its development, and alter the procedure in response to input that would be seen throughout model training. Sixty respondents from the Pasir Jambu subdistrict, Bandung district, and Sugih Mukti village community participated in this survey, with 22 women (38.3%) and 37 males (61.7%) being the majority. The composition of the initial respondents consisted of the following elements: government/village officials, community leaders, health cadres, and youth organizations. According to the survey results, earthquakes (80%), landslides (11.7%), and high winds (8.3%) are the top three disaster hazards that the residents of Sugih Mukti Village feel most at risk from. The examination of knowledge and attitudes about disasters and disaster management produced very unsatisfactory results, with the average knowledge score being 58.75 (SD 12.54) and the average attitude score being 59.31 (SD 10.57). None of the responders could show that they had the necessary abilities or fundamental rescue techniques for catastrophe victims, which were self-performed by the community.

### 2.2. Settings and Participants

The study setting was Marga Mukti Village, Pasir Jambu District, Bandung Regency, West Java, Indonesia. The study was conducted from April to August 2022. The participants were divided into key informants and general informants, with the following inclusion criteria: academics, experts chosen by researchers as conceptual reference materials, indigenous people of the local community who serve as community leaders and have experienced disasters, and traditional stakeholders who are used as reference figures and experts. A total of 4 experts and 11 informants from the defense forces, government officials, community leaders, BNPB, business owners, and professional nurses were selected through a purposive sampling technique.

### 2.3. Data Collection

Data collection was done through in-depth interviews, Focus Group Discussions (FGDs), and expert panel stages in the Indonesian language. In-depth interviews were conducted for 30–60 min, and FGDs were conducted for 60–90 min. Each respondent’s responses were calcified until data saturation was reached. The protocol for preventing the spread of COVID-19 is carried out by the standards established by the Indonesian government, including the use of masks and face shields, establishing a minimum distance of one meter between participants, washing hands with disinfectant before entering and leaving the room, and measuring body temperatures before participants enter the room. The expert panel stages were held virtually by using Zoom for 60 min.

Field notes are made by writing down everything significant that happened while conducting the research, including taking photos of significant areas. The study team conducted the interviews with the aid of two administrative officers who supported the interview procedure. Data saturation was accomplished after the researcher identified repeated answers from research informants with meanings (themes) that lead to the same topic.

### 2.4. Data Analysis

Data analysis is carried out in four stages of analysis, as described by Leininger (2002). The first stage is collecting, describing, and documenting raw data. At this stage, researchers collect data, followed by explaining and documenting the raw data obtained from interviews, FGDs, surveys, and documentation studies. The second stage is the identification and categorization of descriptors and components, specifically, selecting and classifying descriptors and data elements that are the primary study topic. At this point, the emphasis is on identifying the contributing elements and impediments that hinder the creation of a community-based disaster risk-reduction model with a culturally sensitive strategy to enhance community preparedness. The third stage is the pattern and contextual analysis, which identifies patterns of interactions, values, beliefs, and practices when they are related to informants and data gathered through field observations. Identifying the key themes, research findings, and patterns of community engagement in disaster risk-reduction strategies in the community are covered in the fourth stage, which is titled “major themes, research findings, theoretical formulations, and recommendations”. The steps of interpretation and synthesis of results form the foundation at this level.

### 2.5. Ethical Considerations

Ethical approval for this study was granted by the Health Research Ethics Committee of the Ministry of Health, Republic of Indonesia, Bandung Polytechnic of Health (ref. No. 01/KEKP/EC/II/2022). Permission to conduct the study was also obtained from the community leader of Marga Mukti Village, Pasir Jambu District, Bandung Regency, West Java, Indonesia. The voluntary and confidential nature of the study was explained to participants before each interview, Focus Group Discussions (FGDs), and expert panel stages process. To enhance confidentiality, pseudonyms were used in the study.

### 2.6. Trustworthiness

Trustworthiness was completed by identifying research findings evaluated through a process including credibility, confirmability, meaning in context, recurrent patterning, saturation, and transferability. Credibility was established by participating in the daily lives of informants, ongoing observation, triangulation, peer debriefing, and community support groups, community leaders, and social organizations that promote DRR. Confirmability was clarified by stating ideas or findings that were heard, seen, or experienced with key informants and several general informants. In order to interpret and grasp the significance of meaning in context, researchers incorporated the information from interviews, observations, and documents. All key informants and some general informants then corroborated regarding the interpretation. All information was based on contextual reality and the environment. Recurrent patterning was carried out with researchers who used informants’ repeated experiences, expressions, events, or activities in relation to community-based disaster risk reduction. Transferability and saturation were met when the data collected reveal duplication of content related to ideas, meanings, experiences, descriptions, and other similar expressions from the informants or repeated observations. Further research findings were reported in a rich language style, including quotes, comments, and stories that added to the richness of the report and provided for understanding the context of the experience in which it all took place. Researchers attempted to facilitate transferability by providing detailed documentation across all phases of research.

## 3. Results

### 3.1. Document Analysis

Based on the document analysis, Sugihmukti Village is located in Pasir Jambu District, Bandung Regency, West Java Province. This village located in a mountainous highland, with an area of 1767.96 km^2^, at 107.407795 east longitude and −7.19077 south latitude. The distance from the village to the subdistrict capital is as far as 7000 km. Sugih Mukti Village is a disaster-prone village due to its unstable soil structure and is located in an area adjacent to an active volcano and geothermal mining activities in the Patuha Mountains. Previous research was conducted on community representatives involving 60 respondents from two disaster-prone areas in the village of Sugih Mukti. The results reveal that the priority disaster risks that are most experienced by the people of Sugih Mukti Village are earthquakes (80%), landslides (11.7%), and strong winds (8.3%), while the others are fire disasters and conflicts between communities. The results of measuring the level of knowledge, attitude and skill of the community about disaster, are obtained: knowledge, 58.75% (SD 12.54); attitude, 59.31% (SD 10.57); and skills in providing basic health assistance, 0%, indicating the inability to take action. Based on these results, this research was developed.

### 3.2. Expert Discussions and National Seminar

The initial stage of the research began with expert discussions and national seminars with four key informants from BNPB, PPKK-Kemenkes, PPNI, and academics of disaster nursing experts. The results of expert discussions and seminars were identified as central themes in the development of a culturally sensitive Public Health Nursing (PHN)-based disaster risk-reduction preparedness training model. The resulting research themes are as follows.

1. The implementation of the global target achievement of the Sendai Framework in Indonesia is carried out using the Disaster Risk Reduction (DRR) paradigm for the Disaster Resilient Village program.
2. DRR is operated in the form of community empowerment.
3. The steps for community empowerment in disaster management are identical to the community nursing process: assessment, diagnosis, planning, implementation, and evaluation.
4. The PPNI Professional Organization believes that there is a need for community empowerment through guiding community nurses with structured disaster management training.
5. Community-integrated training in disaster preparedness is needed as a DRR effort.

### 3.3. Focus Group Discussions (FGDs)

The next stage was FGDs and in-depth interviews with 11 informants from the defense forces, government officials, community leaders, BNPB, business owners, and professional nurses. The results of FGDs and in-depth interview are us follow (Table 1):

Based on Table 1, five themes are generated which become the basis for developing a preparedness training model for Public Health Nursing. The five themes are:

1. The Sugih Mukti area is prone to disasters due to its geographical structure.
2. No one in the community or its officials have participated in integrated disaster training in the health sector based on community needs.
3. Government efforts have not been effective in community disaster management.
4. A community development model in integrated disaster management that involves all components of society is needed.
5. DRR efforts are not yet optimal

## 4. Discussion

### 4.1. Description of Model Requirements

**Figure 1:**
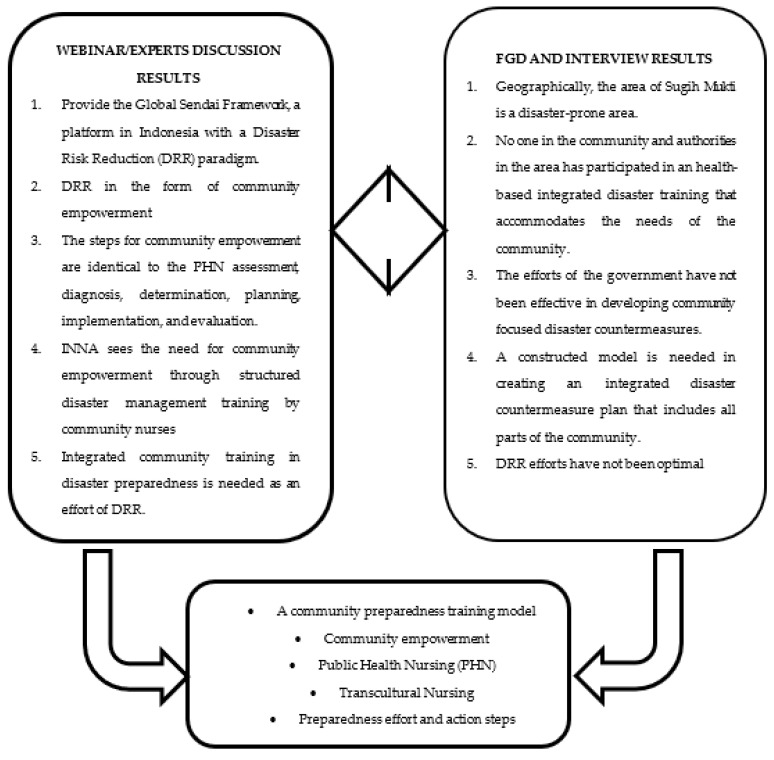
Thematic description of Disaster Risk Reduction (DRR) needs in community nursing.

Figure 1 describes the results found through investigating the need for a Public Health Nursing (PHN) disaster risk-reduction preparedness training model as a guide for community nurses. The model is an integrated implementation of community care and empowerment stages in disaster-prone areas. The thematic exploration results from all community members show that a disaster risk-reduction preparedness training model that is integrated with community empowerment is very much needed.

This need is in line with BNPB’s values, which are preparedness and level of community independence as the most important aspects for disaster management, followed by the help of family members, friends, SAR team, health team, and surrounding components [19]. To strengthen this opinion, the community becomes the focus of studies in disaster risk reduction, which starts from groups of school children and adolescents, health cadres, and community leaders as targets for empowerment in disaster risk reduction. Community empowerment in disaster risk reduction requires a community-centered, interprofessional socialization approach that is systematic, culturally informed, and focuses on modeling the role of trainers, caring for officers as well as attention to the needs of participants [20].

Involving the community in disaster risk-reduction studies will increase the awareness of the community. Research on public health recommended the need for a stronger emphasis on public health in managing disasters. For example, the Reserve Corps provides civil medical training to improve public health infrastructure and provides greater opportunities to collaborate with communities in disaster management [21] Lebowitz (2015). In order to make these various components effective, a model is needed that can accommodate the increasing capacity of the community. According to Paton (2019), facilitating the community’s ability to anticipate, prepare, and recover from disasters is an important component of the UNISDR strategy [15]. Pourvakhshoori et al.’s (2017) research provides clarity that nurses are part of the health profession which has a very important role [22]. 

Understanding the experience of nurses in disasters can help identify problems in the disaster nursing services field, which can be resolved by better planning and preparation [22]. One of the major problems in the disaster management system is the lack of planning to employ and organize volunteers in the health sector during a disaster [23]. The nursing process approach, using the concept of the Transcultural Nursing model, is used as an effort to accelerate the Public Health Nursing (PHN) program, which is in accordance with the government program.

The integrated implementation program of Public Health Nursing in Disaster Risk Reduction (DRR) and the transcultural nursing approach is very relevant to community groups that are socioculturally heterogeneous. In the context of providing holistic service, services with a culturally sensitive approach are needed; nurses must adapt to the system, norms, and culture that apply in a community. 

This is in line with the anthropological view, which believes that cultural factors influence human behavior. When faced with danger, people not only consider the dangers they can face, but also prioritize factors such as social values, religious beliefs, traditions, and attachments to a particular place or location. Culture has the power to increase or decrease a society’s vulnerability to disasters. Lack of consideration of the cultural aspects of the affected communities can hinder effective DRR strategies, thereby increasing the vulnerabilities of the affected communities rather than reducing them. Therefore, culture has the power to increase or reduce community vulnerability to disasters [11] Kulatunga (2010). Research by Kertamuda and Chris (2012) explains that the culture of resignation and patience in the three largest ethnic groups on the island of Java (Betawi, Sundanese, and Javanese) is still very dominant in responding to disasters [24].

### 4.2. Constructed Model Requirements

**Figure 2:**
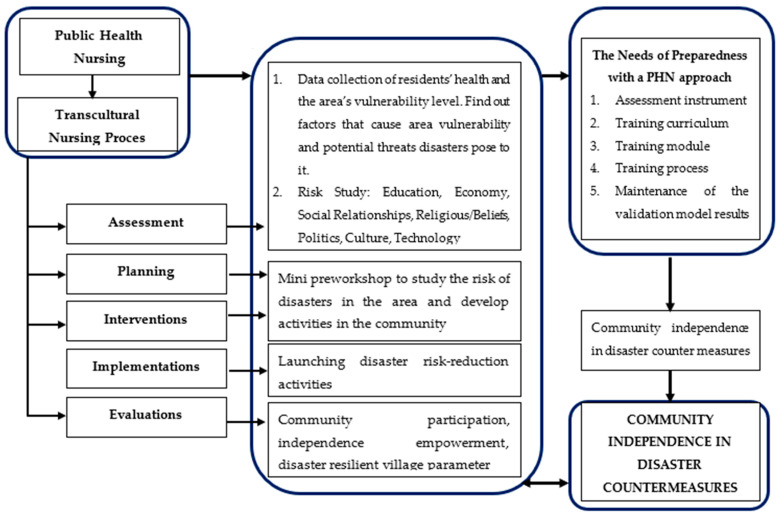
Constructed requirements for the Public Health Care-Based Disaster Preparedness Training integration model.

Figure 2 shows the structure of the model that was built based on the thematic analysis of the need for disaster risk-reduction preparedness training. The training model is constructed from the concept of community nursing theory (PHN) as well as the strengthening of the transcultural nursing model. The steps of community empowerment in disaster management are in line with the steps of the community nursing process, which are assessment, planning, intervention, implementation, and evaluation.

The three types of priority disasters that the people of Sugih Mukti frequently experience are earthquakes, landslides, and hurricanes. This result is similar to the Bandung Regency BPBD report, which identifies the types of priority disasters in Bandung Regency: floods, landslides, cyclones, droughts, and earthquakes. Sugih Mukti Village, Pasir Jambu District, Bandung Regency, is included in the list of areas prone to landslides and earthquakes [25]. This is due to the geographical location of Sugih Mukti Village. It is located within a geothermal exploration area, also known as the Geothermal Patuha Mountain in Bandung district. The activity of the Geodipa Energi project is a leading factor that increases Sugih Mukti Village’s vulnerability to landslides and volcanic earthquakes [26]. Research conducted on the residents reveals that the level of knowledge, attitudes, and skills of the community are still low. Almost all of the people of Mekar Mukti Village stated that they had never received community-based disaster management training. These results are in line with research conducted by Setiawan et al. (2017), which emphasizes the importance of training to empower rural communities living in disaster-prone areas that will normalize the physical and psychological problems of natural disaster victims [9].

The role of the community nurse becomes very important in this situation. Research by Walsh et al. (2012) explains that effective disaster preparedness, response, and recovery requires a well-planned, concerted effort by experienced professionals who can apply specific knowledge and skills in critical situations. The results can provide a useful starting point to identify the level of competence expected of health professionals in disaster medicine and public health [27]. A study by Gulzar et al. (2012) provides results that support this. The intervention, collaboratively chosen by the CHN, was shaped by a planning framework that fits the nursing process. It is a method used to take a holistic approach in the earthquake-affected area. Such a framework includes four phases; assessment, planning, implementation, and evaluation. The framework provides systematic and specific directions for healthcare providers while promoting public health [28].

Communities in disaster-prone areas must have the ability, strength, and independence in managing disasters at every stage. Results from a study link cultural factors with Disaster Reduction Risk (DRR) activities and highlight how culture has influenced DRR activities. In some ways, culture has become one of the factors of community survival from disasters. Therefore, it can be said that culture has the power to increase or decrease people’s vulnerability to disasters [11]. An expert opinion, Rush et al. (2019), explain that disasters can occur in any community at any time. Such an opinion becomes true in any disaster. Nurses have a central role in managing and preparing for medical care during these catastrophic episodes [29]. Study results from Salmani et al. (2019) show the importance of having working management. It is necessary to have complete legislation, NGOs, and sociocultural factors, preparedness, response, retention, relocation, termination, and follow-up in each nurse’s role in disaster-prone areas [23]. 

However, not all nursing roles can be easily implemented in disaster situations. A study by Ahmadi et al. (2018) found that the elders of a community that went through a disaster faced various challenges in their everyday life, which means that more efforts were needed to help them reach the stage of recovery. Taking into account these conditions, the need for community empowerment during the disaster period must not be postponed. Training the community to be independent must be done constantly by taking into account local cultural conditions [30]. The results of research in China show several conditions that push the need for community empowerment in disaster locations, which follow [20]. The behavior of the trainers influences the participants through their interactions during the training process. A mental health training program is needed to identify the needs of disaster workers and victims. Building a systematic interprofessional education strategy is required. Systematic interprofessional education can assist in responding to complex local problems due to a collaborative approach because it bridges the gap between theory and practice, and solves local needs and international guidelines.

Training regulations are needed to maintain and monitor the quality of the content, standards, ethics, and codes of conduct at all levels. A community-centered interprofessional education approach is required. It focuses on modeling the role of trainers, caring for staff, attending to the needs of trainees, and building a systematic, culturally informed, and informed interprofessional education strategy. 

The results of the study by Lebowitz (2015) supported this opinion, regarding the need for a stronger emphasis on public health workers in managing disasters. For example, civil medical training for reserve corps can be done to improve public health infrastructure and provide greater opportunities to collaborate with the community in handling disasters [21].

Raising awareness through disaster education and socialization is very necessary. By doing so, everyone can understand risks, be able to manage threats, and, in turn, contribute to encouraging community resilience from the threat of disaster. Additionally, social cohesion, collaboration, and mutual trust are the adhesive values of the society that have been nurtured, both individually and collectively by the community, to prepare for, respond to, and rise from adversity caused by disasters.

## 5. Limitation

This research is limited since the model has not been tested or used in this study, and its effectiveness has not been determined. During the following stage of the research, the model will be tested and used. Additionally, obtaining data relating to culturally sensitive features and local strengths has not been thoroughly investigated, and requires a more thorough investigation in future research.

## 6. Conclusions

The study findings highlight the importance to the community of the Disaster Risk-Reduction Preparedness Model Based on Culturally Sensitive Public Health Nursing. The community is approached using a combination of the Public Health Nursing (PHN) and Transcultural Nursing principles. An integrated model for disaster preparedness training based on public healthcare as a solution to empower communities in managing disaster risk is validated, constructed, and designed. This model consists of five main components: nursing care assessment instruments and introductory surveys for disaster-prone communities; public health nursing-based disaster preparedness training integration curriculum; public health nursing-based disaster preparedness training module integration training process; public health nursing-based disaster preparedness training process; and maintenance of training results.

## Figures and Tables

**Table 1:** Qualitative data of the FGD results.

Data	Subthemes	Theme
“… The people of Sugih Mukti Village often experience disasters. Especially landslides and tropical cyclones” (Respondents: 1, 2, 4, 5, 10) “Well, we’re used to it, sir. Happens very often. Especially if you’ve heard the roar from the Geo Dipa Gas Production” (Respondents: 4, 5, 6) “The location is indeed close to the drilling center, not the production process itself but very close to Patuha Mountain, which makes small earthquakes and has volcanic activity” (Respondents: 1, 2, 3, 10) “… South Bandung Regency is prone to disasters because of geological and geographical factors…” (Respondents: 1, 2, 6, 10) “often sir, even though I’m new, we’re already used to the vibrations, not because of Geodipa,… Geodipa wants to help” (Respondents: 6, 7, 11) “We know the risks, but we just do not care, the government has thought of us, if we move where will we go?” (Respondents: 5, 11) “Once, sir, there was a hurricane, there were people whose houses were partially damaged, but most often there were landslides” (Respondents: 5, 11) “The hardest thing, sir, was a flash flood, sir, there were many victims, there were 13. Wow, at that time it was like the end of the world, I was not yet a village official, but I was bewildered, sir, the people were suffering” (Respondents: 4, 11)	Disasters that often happen: constant landslides; A big flash flood in 2013; Whirlwinds and volcanic earthquakes all the time; The location is close to the Patuha volcano and Geodipa Ltd., a natural gas production center.	Geographically, the Sugih Mukti area is prone to natural disasters
“I don’t know what to do if there is a landslide or flood, maybe because my house is far away, but other people also don’t know yet. Those who know if an earthquake is happening run away” (Resp: 4, 5, 11) “As government officials, we are always ready for orders, instructions from commanders, there are ‘babinsa’ (army officers) and village police who monitor general security, but if we are needed, we often go down ” (Resp: 1, 2, 3, 4, 6, 10) “The residents are only focused on finding a way to get food, sir, they don’t understand how to fight a disaster, if there’s a flood we flee, if there we hear rumbling noises we just let it go, the most important thing is to pray” (Resp: 4, 5, 11) “The government has tried to explain to villages, maybe not sugih mukti village, but other parties have done it, we will help and support” (Respondents: 4, 6, 10) “There are difficulties, sir, in educating the public like money, methods and government support, as well as those who have money… well the company. In order for them to want to help, they must be given consumption, food, transportation, etc.” (Respondents: 4, 5, 6, 9, 10) “As I see it, it’s not optimal yet. We are only limited to the health sector through cadres. As for disasters, we haven’t been able to do it” (Resp: 7, 8, 10) “it’s very important, sir, we are even ready to learn, to help, after all I guide this area” (Resp: 1, 2, 7, 8) “It’s time to do more. For example, COVID, sir. For the residents, it’s normal. If you get a fever, well you just compress your forehead with cloth, you lose your sense of smell, ah, just take village medicine, we give them bandrek, or a potion, no one wears a mask, so there’s no need, the most important thing is if you are able to eat” (Respondents: 6, 11) “The government officials are the ones who are the most responsible, the people are the citizens, we just follow the government’s advice” (Respondents: 5, 11) “We will support, we know that the community must be involved, that’s why I like coordinating with villages, MSMEs, clinics, and treatments at the public health center, we will help” (Respondents: 7, 8)	People do not think there is a problem in terms of disaster; Sectoral support has not been managed properly; The government’s efforts have been carried out but have not been effective; Lack of coordination in disaster management involving the community and industry; The readiness of the industry to assist and support disaster prevention and management through CSR.	PRB efforts have not been optimal
“As health workers in the community, we certainly need things like this, to train the community with disaster management, in particular” (Respondents: 7, 10) “In the community, it is very necessary. In our hospital there is a Hospital Disaster Plan (HDP) for nurses and all health workers, so they can contribute. We don’t know the HDP training method or model at the hospital yet” (Respondents: 10) “Don’t know how, need to be told how to do it or be trained, sir. So that people know. The village is ready to support…” (Respondents: 4, 5, 6, 11) “Someone has to start sir. We are ready to help. We don’t know the models and methods of training our society. Especially in the health sector. There should be guidelines so that people can be educated ” (Respondents: 7, 8, 6, 11) “In the army, there is training for soldiers, sir. If we train people, we don’t know the method yet. If using the military route is not suitable, we are ready to help and cooperate with the public health center and other health care providers” (Respondents: 1, 2, 6) “If BPBD often conducts community training, it is only selective, not for all groups, just certain villages. It’s about the budget too. Those who have been trained are in Cicalengka and Majalya. South Bandung has not been done. Every year there are only enough for 2 villages. The name of the program is Destana” (Respondents: 3, 7) “As far as the health sector is concerned, there is no such thing, I haven’t heard of it” (Respondents: 3, 7, 8, 9, 10) “Nurses are not optimal, sir, because they have a lot of activities. Those who participate in the training are also limited, there are none in the community health centers yet, we have not participated in the community development training in the field of disaster, we still have to continue with other programs” (Respondents: 9, 10) “Community Nurses are too busy with administrative work, sir, this disaster problem has not been addressed, there must be training first, so that we know how far we can train the community” (Respondents: 9, 10)	Health workers have not received information related to disaster care in the community; There is no model that can be used as a guide in educating the public; Cross-sectoral coordination is not yet optimal—(TNI/Polri/Government) do not know the guidelines for community development in the field of disaster management planning; The community needs guidance for disaster management from a health worker.	There needs to be a model of community development in disaster management
“The government’s efforts have not gone well, for example the need for heavy equipment for landslides, it is difficult and takes time if needed” (Respondents: 1, 2, 3, 11) “The government has been good. The Health Office has coordinated to meet the needs, it just needs more organization. For example, referrals to hospitals have not been optimal, difficulty in costs, treatment requirements. Because of this, there are victims who are rejected” (Respondents: 9, 10, 7) “People usually struggle when there is a disaster. Help is lacking and late and we are the ones who are blamed, sir. Especially if there are health problems that we don’t know what to do” (Respondents: 4, 5, 11) “I’m not sure about the government, but us as community partners are responsible. We will help if there is an institution that will develop the community, like now. we are ready to cooperate if needed” (Respondents: 7, 8, 11) “The government has tried, there are many programs for disasters. For example, for COVID 19. This is a disaster too; it just can’t be solved as fast as turning the palm of the hand. Moreover, the community has different characters, now the government has tried very hard, the sub-district always coordinates with BPBD to map if there are locations that are threatened with landslides” (Respondents: 3, 6) “For the wider community, it has not been felt, sir, the government likes to be unfair, in other places it is fast, while in our place it takes a long time. I don’t know why, the fast ones are the army, sir. Maybe it’s because there are noncommissioned law enforcement officers, huh… And the police take action if there is a report” (Respondents: 4, 5, 11) “At BPBD, we try to maximize our efforts, sir, but it’s only limited to human resources. For example, if there is a health problem, we coordinate with the service, we cannot handle it directly. BPBD can only coordinate. That’s right, sir, we can’t do it all, especially human resources. We don’t have all professions, so we really need cooperation” (Respondents: 3)	Need cooperation and coordination; Health coordination model should be developed; People have not fully felt the presence of the government; The program exists, but it is not yet operational.	Government efforts have not been effective
“I have never participated in any training, and indeed there has never been any in the village for our community, especially those related to the health sector” (Respondents: 4, 5, 6, 7, 10, 11) “ If we have not participated in disaster management training, and in the Army, the training is the same as military training. If you want to go to the area there has to be a special course on how to approach the community. Just not specialized in disaster management training” (Respondents: 1, 2) “As a BPBD extension worker, I have often participated in training, but I have never attended training in the community that was held by health workers. The most frequent, yes.. training with ‘PT” (a company), but the materials are usually trauma healing, soup kitchens, rarely about health…” (Respondents: 3) “To my knowledge in our area there has never been any special disaster management training for community members, including by BPBD. I feel like it’s never been done. So if the Polytechnic of Health wants to hold it, we will support and help” (Respondents: 7, 8, 6) “ I was once invited by the Geodipa representative for disaster counseling in Ring 1, our area, only so far it has not been held, but I heard that Geodipa held it themselves without coordination with us. That we don’t know for sure” (Respondents: 4) “If it is true that there will be training in our area, we have been waiting for this, actually this is included in our program too, especially in villages that are in direct contact with the mountain and gas production center, indeed there has been compensation, but there has never been training and counseling about disasters” (Respondents: 5, 6) “ In our area, there is no cadre training for nurses, let alone for the community. So we have never guided the community for disaster management, in the future, if God wills it, we will program it” (Respondents: 7, 10) “ I work at the hospital and the Disaster Hospital Plan training had been done, specifically with the emergency room team and the disaster team in each hospital, but for the surrounding community, we at the hospital have never held it” (Respondents: 9)	The community has not been involved in integrative disaster training; BPBD, TNI, Polri, village, and subdistrict governments do not know the integrated disaster training model for the community.	No involvement yet in an integrated disaster training program in the health sector

## Data Availability

Not applicable.
